# Bromoenol Lactone, an Inhibitor of Calcium-Independent Phospholipase A_2_, Suppresses Carrageenan-Induced Prostaglandin Production and Hyperalgesia in Rat Hind Paw

**DOI:** 10.1155/2015/605727

**Published:** 2015-04-30

**Authors:** Keiichiro Tsuchida, Takae Ibuki, Kiyoshi Matsumura

**Affiliations:** ^1^Department of Anesthesiology, Graduate School of Medical Science, Kyoto Prefectural University of Medicine, Kamigyo-ku, Kyoto 602-8566, Japan; ^2^Department of Biomedical Engineering, Osaka Institute of Technology, Asahi-ku, Osaka 535-8585, Japan

## Abstract

Prostaglandin (PG) E_2_ and PGI_2_ are essential to hyperalgesia in inflammatory tissues. These prostaglandins are produced from arachidonic acid, which is cleaved from membrane phospholipids by the action of phospholipase A_2_ (PLA_2_). Which isozyme of PLA_2_ is responsible for the cleavage of arachidonic acid and the production of prostaglandins essential to inflammation-induced hyperalgesia is not clear. In this study, we examined the effects of two PLA_2_ isozyme-specific inhibitors on carrageenan-induced production of PGE_2_ and PGI_2_ in rat hind paw and behavioral nociceptive response to radiant heat. Local administration of bromoenol lactone (BEL), an inhibitor of calcium-independent PLA_2_ (iPLA_2_), significantly reduced carrageenan-induced elevation of prostaglandins in the inflamed foot pad 3 h after injection. It also ameliorated the hyperalgesic response between 1 h and 3 h after carrageenan injection. On the other hand, AACOCF_3_, an inhibitor of cytosolic PLA_2_, suppressed neither prostaglandin production nor the hyperalgesic response. BEL did not suppress the mRNA levels of iPLA_2_ *β*, iPLA_2_ *γ*, cyclooxygenase-2, microsomal prostaglandin E synthase, prostaglandin I synthase, or proinflammatory cytokines in the inflamed foot pad, indicating that BEL did not suppress inflammation itself. These results suggest that iPLA_2_ is involved in the production of prostaglandins and hyperalgesia at the inflammatory loci.

## 1. Introduction

Acute inflammation produces a condition known as hyperalgesia, which is characterized by enhanced pain sensation and reduced pain threshold. This abnormal sensory state is brought about, at least in part, by sensitization of peripheral nociceptors. Accumulating evidence indicates that inflammatory mediators including prostaglandin E_2_ (PGE_2_) and PGI_2_ are responsible for the sensitization of nociceptors [1, 2]. Recent studies further clarified the molecular mechanism of sensitization: PGE_2_ and PGI_2_ enhance or sensitize the activity of the capsaicin receptor transient potential receptor V1 (TRPV1) through the activation of PGE_2_ EP1 and PGI_2_ receptors, respectively [3]. Protein kinase C and protein kinase A are involved in the activation of TRPV1 by these prostaglandins. In contrast, cyclopentenone prostaglandins, including 15-d-PGJ_2_, PGA_2_, and PGA_1_, which are metabolites of PGD_2_, PGE_2_, and PGE_1_, respectively, directly activate the irritant receptor TRPA1 [4]. For pharmacological control of inflammatory hyperalgesia, it is important to understand how these prostaglandins are produced in inflamed tissues.

Prostaglandins are generated through the arachidonic acid cascade [5], which involves three enzymatic steps: first phospholipase A_2_ (PLA_2_) cleaves membrane phospholipids and releases arachidonic acid; second, cyclooxygenase (COX) converts arachidonic acid into PGH_2_; and, third, various types of prostaglandin isomerases convert PGH_2_ to bioactive prostaglandins, including PGE_2_ and PGI_2_. Each enzymatic reaction can involve multiple isozymes. Recent studies have shown that COX-2, an inducible isozyme of COX, and microsomal prostaglandin synthase-1 (mPGES-1), an inducible isozyme of PGE synthase, are responsible for the generation of PGE_2_ in inflamed tissues [6, 7]. In these studies, administration of a COX-2 inhibitor to rats [7] or disruption of the mPGES-1 gene in mice lowered PGE_2_ content in inflamed tissues and eased pain-related behaviors [6]. Thus, COX-2 and mPGES-1 could be pharmacological targets for the treatment of inflammatory pain.

However, it is not clear which isozyme of PLA_2_ is responsible for the production of prostaglandins and development of hyperalgesia in inflamed tissues. PLA_2_ has over ten isozymes which are classified into three categories based on their structural and functional similarities [8, 9]. First, cytosolic PLA_2_s (cPLA_2_ or group IV PLA_2_) are located in the cytoplasm and are activated by low calcium ion concentrations (*μ*M levels). Second, secretory PLA_2_s (sPLA_2_ or group IB, II, V, or X PLA_2_) are released into the extracellular space where they are activated by high concentrations (mM levels) of calcium ions. Third, calcium-independent PLA_2_s (iPLA_2_ or group VI PLA_2_) are present in the cytoplasm and do not require calcium ions for their enzymatic activity, although the precise mechanisms for activation are unclear. Among these PLA_2_s, a significant role for iPLA_2_ in the production of prostaglandins in carrageenan-induced pleuritis in rats was demonstrated [10]. It is also reported that, in the spinal cord, cPLA_2_ seems to be involved in inflammation-induced hyperalgesia [11]. In the present study, we examined if iPLA_2_ and/or cPLA_2_ are responsible for the production of prostaglandins and development of hyperalgesia in carrageenan-induced inflammation in the rat hind paw.

## 2. Materials and Methods

### 2.1. Materials

Male Sprague-Dawley rats (nine weeks old, 300–320 g) were purchased from Charles River Laboratories (Yokohama, Japan). All chemicals were obtained from commercial suppliers: bromoenol lactone (BEL; iPLA_2_ inhibitor), arachidonyl trifluoromethyl ketone (AACOCF_3_; cPLA_2_ inhibitor), PGE_2_ enzyme immunoassay (EIA) kit, and 6-keto-PGF_1*α*_ EIA kit from Cayman Chemical (Ann Arbor, MI); Isogen (RNA extraction solution) from Nippon Gene (Tokyo, Japan); reverse transcription Kit from Invitrogen (Carlsbad, CA); PCR Sybr Green master mix, LightCycler TaqMan Master, and TaqMan Probes from Roche Diagnostics (Indianapolis, IN); and RNAlater (RNA stabilization solution) from Ambion (Austin, TX).

### 2.2. Animals

All experiments were carried out according to protocols approved by the Institutional Animal Care Committee of Kyoto Prefectural University of Medicine. Rats were housed four per cage and maintained on a 12 h light/dark cycle (light on 8:00–20:00) with controlled temperature (25 ± 3°C) and humidity (55 ± 15%). Animals were allowed free access to food and water at all times.

### 2.3. Pharmacological Treatment

The plantar surface of the left paw received a subcutaneous injection of either 3 mg type *λ* carrageenan (Sigma-Aldrich, St. Louis, MO) dissolved in 100 *μ*L saline or saline alone. Just after carrageenan injection, one of the two PLA_2_ inhibitors (30 nmol) or vehicle (100 *μ*L of 0.1% dimethyl sulfoxide (DMSO) in saline) was injected into the same site. The inhibitor dose was determined according to the study by Gilroy et al. [10]. For biochemical analyses, each rat was anesthetized with pentobarbital (60 mg/kg, intraperitoneal injection) 3 h after subcutaneous injection, and the left hind leg was cut at the knee, quickly frozen in dry ice powder, and kept at −80°C until further processing. Rats were killed by decapitation. This time point was determined based on the following nociceptive behavior study, in which a PLA_2_ inhibitor was effective between 1 h and 3 h after injection.

For the assessment of nociceptive behavior response, rats were injected with the carrageenan/saline and PLA_2_ inhibitors/vehicle in the manner described above. Thermally evoked paw-withdrawal response was assessed using a device developed in Yaksh's lab [12]. The paw-withdrawal latency was measured for the left hind paw before and every 60 minutes after the subcutaneous injection for a total of 360 minutes. For each time point, the latencies were measured 3 times in each rat and averaged. Paw-withdrawal latencies were expressed as ratios to the baseline value of each rat.

### 2.4. Biochemical Assay

For analyses of PGE_2_ and 6-keto-PGF_1*α*_ (a metabolite of PGI_2_), the hind paws were coronally cut into 50 *μ*m thick frozen sections in a cryostat at −20°C. Twenty of these sections were collected in a plastic tube containing precooled ethanol (1 mL) and indomethacin (10 *μ*g) which prevents the synthesis of prostaglandins during tissue processing. After measuring wet tissue weight, the sections were homogenized with polytron for 30 s followed by sonication for 20 s. The homogenates were centrifuged (15000 rpm for 20 min at 4°C) and the supernatant was collected. Ethanol was evaporated from the supernatant using a vacuum centrifuge. Prostaglandins, which were retained in the tube, were dissolved in EIA buffer. PGE_2_ and 6-keto-PGF_1*α*_ were measured using EIA kits according to the manufacturer's instructions. Tissue pellet remaining in the plastic tube was heated in a heat block to completely evaporate the ethanol. The weight of dried pellet was considered to be the dry tissue weight of the paw from which the prostaglandins were extracted.

### 2.5. Real-Time RT-PCR

Frozen paw sections were prepared as described above. Twenty of these sections were placed into a vial containing RNA later (1 mL) and stored at −30°C until further processing. For RNA extraction, the samples were homogenized in 1 mL phenol-based RNA extraction solution (Isogen) with polytron for 30 s followed by sonication for 20 s. Total RNA was isolated according to the manufacturer's instructions. cDNA was prepared from total RNA using M-MLV reverse transcriptase and random hexamer as the primer. The reverse-transcribed cDNA was amplified using a light cycler (Roche Diagnostics). mRNAs of COX-2, mPGES-1, iPLA_2_ *β*, iPLA_2_ *γ*, and GAPDH were quantified with the Sybr Green protocol. For the quantification of mRNAs of prostaglandin I synthase (PGIS), interleukin-1*β* (IL1*β*), and interleukin-6 (IL6), the TaqMan Probe protocol was used. Primer pairs used for the PCR reaction were as follows:

 COX-2: 5′-ctcactttgagtcattc-3′, 5′-gattagtactgtagggttaatg-3′,
 mPGES-1: 5′-aatgaacccacgcattcgct-3′, 5′-cagccttcatggctccgtct-3′,
 iPLA_2_ *β*: 5′-caaggaactgggcaagatgg-3′, 5′-agagggcgttgaccagcact-3′,
 iPLA_2_ *γ*: 5′-gaataccacaacatacacga-3′, 5′-acctaaaatacgtgtcagca-3′,
 GAPDH: 5′-tgaacgggaagctcactgg-3′, 5′-tccaccaccctgttgctgta-3′,
 PGIS: 5′-atgccatcaacagcatcaaa-3′, 5′-gctccaggtcgaaatgagtc-3′, TaqMan Probe (UPL #18),
 IL1*β*: 5′-tgtgatgaaagacggcacac-3′, 5′-cttcttctttgggtattgtttgg-3′, TaqMan Probe (UPL #78),
 IL6: 5′-cccttcaggaacagctatgaa-3′, 5′-acaacatcagtcccaagaagg-3′, TaqMan Probe (UPL #20),
 GAPDH: 5′-agctggtcatcaatgggaaa-3′, 5′-atttgatgttagcgggatcg-3′, TaqMan Probe (UPL #9).

Relative expression levels of each gene were calculated with the following formula:

(1) Relative  expression  of  mRNA=2cycle⁡  number  of  GAPDH2cycle⁡  number  of  gene  of  interest.

Specificity of the PCR products was checked by the melting curve and by agarose gel electrophoresis in the Sybr Green protocol.

### 2.6. Statistics

The significance of differences between two groups was determined by* t*-test. The significance of differences among multiple groups was determined by multiple* t*-tests with Bonferroni correction. Differences were considered significant at *P* < 0.05. Data are presented as mean ± SEM.

## 3. Results

**Figure 1:**
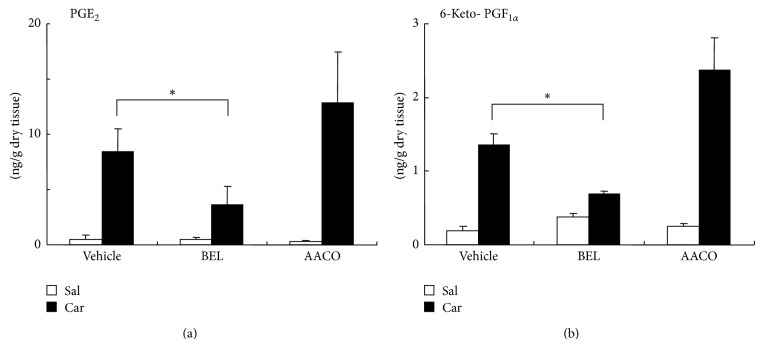
Contents of PGE_2_ (a) and 6-keto-PGF_1*α*_ (b) in rat hind paw. Carrageenan (Car) injection (filled bars) significantly elevated both prostaglandin levels compared to saline (Sal) injection (open bars) in vehicle-, BEL-, and AACOCF_3_- (AACO-) treated groups (*P* = 0.0002–0.014,* t*-test, *N* = 4 in each group). BEL but not AACOCF_3_ significantly suppressed carrageenan-induced increases in PGE_2_ (a) and 6-keto-PGF_1*α*_ (b) compared to vehicle (*P* = 0.009 for PGE_2_ and *P* = 0.017 for 6-keto-PGF_1*α*_, multiple* t*-tests with Bonferroni correction, 2 comparisons among 3 groups; vehicle versus BEL and vehicle versus AACOCF_3_). Asterisks indicate the statistical significance of the difference between vehicle + carrageenan group and BEL + carrageenan group.

We examined the effects of PLA_2_ inhibitors on PGE_2_ and 6-keto-PGF_1*α*_ (a metabolite of PGI_2_) levels in inflamed foot pad. Carrageenan and PLA_2_ inhibitors/vehicle were injected into the right foot pad at the same time. Three hours after the injection, carrageenan significantly elevated PGE_2_ and 6-keto-PGF_1*α*_ levels compared to injection of saline alone in vehicle-, BEL- and AACOCF_3_-coinjected groups (*N* = 4 in each group, *P* = 0.0002–0.014) (Figure 1). BEL, an iPLA_2_ inhibitor, significantly suppressed carrageenan-induced increases in PGE_2_ by 57% (*P* = 0.009) and 6-keto-PGF_1*α*_ by 49% (*P* = 0.017) compared to vehicle. On the other hand, AACOCF_3_, a cPLA_2_ inhibitor and less potent iPLA_2_ inhibitor, did not suppress the prostaglandin levels compared to the vehicle-treated rats. The two inhibitors did not exert significant effects on the prostaglandin levels in the saline-injected foot pad.

**Figure 2:**
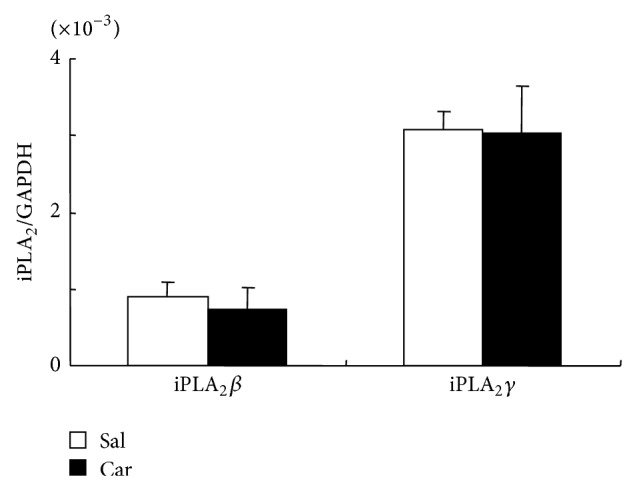
Levels of mRNA of iPLA_2_ *β* and iPLA_2_ *γ* relative to that of an internal control gene (GAPDH) in rat hind paw. Their relative levels were not influenced by carrageenan-induced inflammation. Open bars and filled bars represent results from saline (Sal)-injected group and carrageenan (Car)-injected group, respectively. *N* = 4 in each group.

Real-time RT-PCR studies revealed that mRNAs of both iPLA_2_ isozymes, that is, iPLA_2_ *β* and iPLA_2_ *γ*, were present in the noninflamed foot pad, and their levels did not change following carrageenan injection (Figure 2).

**Figure 3:**
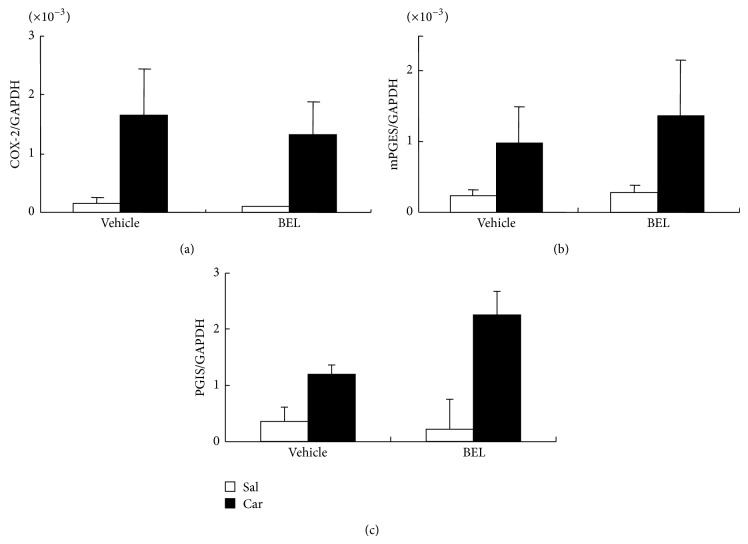
Relative expression of COX-2 mRNA (a), mPGES-1 mRNA (b), and PGIS mRNA (c) in rat hind paw. Carrageenan (Car) injection (filled bars) elevated COX-2 mRNA and mPGES-1 mRNA levels compared to saline (Sal) injection (open bars) though the elevations were not statistically significant by* t*-test except for COX-2 and PGIS in BEL-treated groups. BEL did not significantly influence COX-2 mRNA, mPGES-1 mRNA, or PGIS mRNA levels.

**Figure 4:**
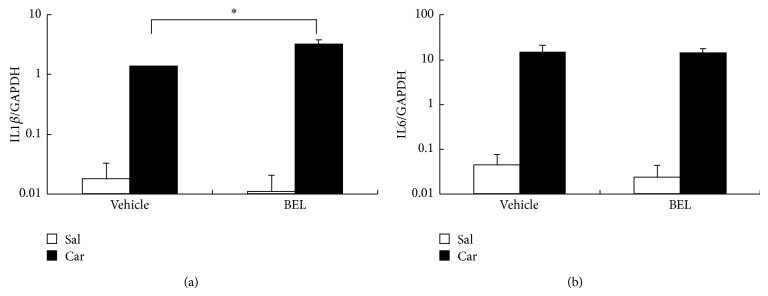
Relative expression of IL1*β* mRNA (a) and IL6 mRNA (b) in rat hind paw. Carrageenan (Car) injection (filled bars) significantly elevated IL1*β* mRNA and IL6 mRNA levels compared to saline (Sal) injection (open bars) (*P* < 0.05). BEL slightly but significantly elevated carrageenan-induced IL1*β* mRNA (*P* < 0.05) and did not affect carrageenan-induced IL6 mRNA.

We then asked if BEL influences the induction of COX-2, mPGES-1, and PGIS, which are possibly involved in carrageenan-induced prostaglandin synthesis. COX-2 mRNA, mPGES-1 mRNA, and PGIS mRNA were increased by carrageenan injection though the increases did not reach the statistically significant level except for COX-2 mRNA and PGIS mRNA in carrageenan + BEL group (Figure 3). There was no significant effect of BEL on the COX-2 mRNA, mPGES-1 mRNA, or PGIS mRNA levels (Figure 3). We also examined the effects of BEL on carrageenan-induced proinflammatory cytokine mRNAs (Figure 4). Carrageenan significantly elevated the mRNA levels of IL1*β* and IL6. BEL slightly but significantly elevated carrageenan-induced IL1*β* mRNA and did not change carrageenan-induced IL6 mRNA. Therefore, BEL seemed to act solely on the enzyme activity of iPLA_2_s resulting in suppression of prostaglandin levels in inflamed tissue.

**Figure 5:**
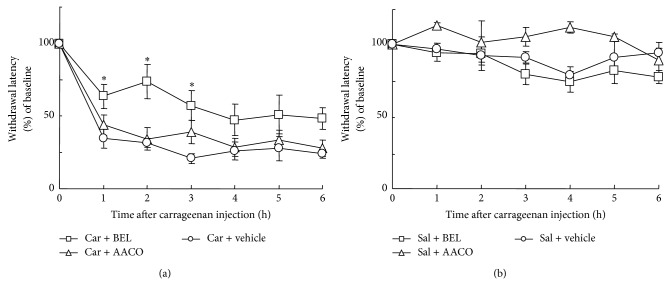
Paw-withdrawal latencies to radiant heat. (a) The latencies were reduced by approximately 70% after carrageenan (Car) injection in vehicle-treated group. The reduction was statistically significant throughout the observation period of 6 h (*N* = 5, *P* < 0.005, multiple* t*-tests with Bonferroni correction, 6 comparisons among 7 groups; time point 0 versus time points 1–6). Treatment with BEL but not with AACOCF_3_ prolonged the latencies at 1 h, 2 h, and 3 h after injection. At these time points, the effect of BEL was statistically significant (*P* = 0.026, 0.014, and 0.015, resp., multiple* t*-tests with Bonferroni correction, 2 comparisons among 3 groups; vehicle versus BEL and vehicle versus AACOCF_3_ in each time point). Asterisks indicate the statistical significance of the difference between vehicle + carrageenan group and BEL + carrageenan group. (b) Saline (Sal) injection had little effect on paw-withdrawal latencies. PLA_2_ inhibitors did not influence the latencies in saline-injected groups.

Lastly, we studied the effects of PLA_2_ inhibitors on carrageenan-induced thermal hyperalgesia. When carrageenan was injected with vehicle, foot withdrawal latency to radiant heat was reduced by approximately 70% compared to the preinjection level (at time point 0), indicating the induction of thermal hyperalgesia (Figure 5(a)). The thermal hyperalgesia was statistically significant throughout the observation period of 6 h (*N* = 5, *P* < 0.005). Carrageenan-induced thermal hyperalgesia was ameliorated by BEL by 44% at 1 h, 61% at 2 h, and 46% at 3 h after injection compared to vehicle-treated group. At these 3 time points, the difference between carrageenan + BEL group (*N* = 6) and carrageenan + vehicle group (*N* = 5) was statistically significant (*P* = 0.026, 0.014, and 0.015, resp.). On the other hand, AACOCF_3_ did not influence the thermal hyperalgesia (Figure 5(a)). Injection of saline did not induce thermal hyperalgesia (Figure 5(b)). Both BEL and AACOCF_3_ did not show significant effect in saline-injected foot pad.

## 4. Discussion

PGE_2_ and PGI_2_ are known to sensitize nociceptors and develop hyperalgesia during inflammation [1, 2]. However, which isoform of PLA_2_ is involved in the synthesis of these prostaglandins in inflammatory tissues was unclear. In the present study, we showed that BEL, a potent inhibitor of iPLA_2_, lowered these prostaglandins by approximately 50% in rat foot pads that were inflamed with carrageenan. The inhibitor also halved carrageenan-induced thermal hyperalgesia during the first 3 h after injection. We confirmed that mRNAs of iPLA_2_ *β* and iPLA_2_ *γ* were constitutively expressed in the rat foot pad. This result is in line with the study by Gilroy et al. [10], who demonstrated that BEL reduced PGE_2_ content in the cell-free inflammatory exudates of the pleural cavity 3 h after carrageenan injection.

It should be noted that BEL is also a potent inhibitor of phosphatidic acid phosphohydrolase. Grkovich et al. [13] reported that BEL suppressed COX-2 expression in LPS-activated macrophages through the inhibition of phosphatidic acid phosphohydrolase-1. Therefore, we wondered if BEL might have reduced prostaglandin levels by suppressing COX-2 induction. However, in the present study, carrageenan-induced COX-2 mRNA was not affected by BEL. This was also the case for mRNAs of mPGES-1 and PGIS, the final enzymes in PGE_2_ and PGI_2_ synthesis, respectively. In line with these results, BEL did not reduce carrageenan-induced proinflammatory cytokines, which are strong inducers of COX-2 and other enzymes in the arachidonic acid cascade. These results strongly suggest that BEL did not significantly suppress inflammation but inhibited iPLA_2_ enzyme activity to reduce carrageenan-induced PGE_2_ and PGI_2_ synthesis and, consequently, thermal hyperalgesia. Therefore, we conclude that iPLA_2_ is responsible for the synthesis of prostaglandins in carrageenan-induced tissue inflammation.

Nonsteroidal anti-inflammatory drugs (NSAIDs) are widely used for the treatment of inflammatory pain. NSAIDs potently inhibit COX but do not suppress the supply of its substrate, that is, arachidonic acid. Thus, free arachidonic acid may enter lipoxygenase or epoxygenase pathways to be various potent eicosanoids such as leukotrienes. This may result in side effects of NSAIDs. On the other hand, since inhibitors of PLA_2_, such as BEL, reduce the supply of free arachidonic acid, PLA_2_ inhibitors may be more ideal in the treatment of symptoms induced by eicosanoids than NSAIDs.

The inhibition of prostaglandin synthesis by BEL was partial—approximately 50% of the control (vehicle alone). This fact suggests that other PLA_2_ isoforms, that is, cPLA_2_ or sPLA_2_s, also participated in carrageenan-induced prostaglandin synthesis. Traditionally, cPLA_2_ has been considered the major PLA_2_ that cleaves arachidonic acid from the sn-2 position of membrane phospholipids during inflammation. In the present study injection of AACOCF_3_, a cPLA_2_ inhibitor, into the foot pad neither reduced PGE_2_ and PGI_2_ nor suppressed thermal hyperalgesia, suggesting that cPLA_2_ does not play a significant role in these responses. This result is consistent with those of two previous studies, the first of which by Gilroy et al. [10] showed no effect of AACOCF_3_ on the PGE_2_ content of cell-free inflammatory exudates of the pleural cavity 3 h after carrageenan injection. The second study by Lucas et al. [11] reported that intraplantar injection of AACOCF_3_ did not suppress carrageenan-induced thermal hyperalgesia even with a dose approximately 20 times higher than that used in the present study. On the other hand, it is reported that a new type of cPLA_2_ inhibitor reduced carrageenan-induced PGE_2_ production in the rat air pouch [14]. The reason for the discrepancy is unclear at present, and we cannot completely exclude the possibility that cPLA_2_ is partly involved in PGE_2_ and PGI_2_ production in inflammatory tissues.

PGE_2_ and possibly PGI_2_ augment nociceptive responses by acting in multiple sites along the pain processing neural pathway. In each site, distinct isoforms of PLA_2_ seem to be involved in prostaglandin synthesis. In the peripheral inflammatory site, these prostaglandins are produced by the action of iPLA_2_ and sensitize nociceptors, as shown in this study. In the spinal cord, PGE_2_ is produced in response to nociceptive neural inputs from the primary sensory neurons and possibly enhances the pain signal transmission [15]. In this site, cPLA_2_ and sPLA_2_ but not iPLA_2_ are reported to play major roles in PGE_2_ synthesis and hyperalgesic response [11, 16]. Furthermore, in the spinal cord and brain, peripheral inflammation induces prostaglandin synthesis through the action of circulating inflammatory cytokines such as interleukin-6 [17]. This is evidenced by an elevation of PGE_2_ in the cerebrospinal fluid and induction of COX-2 in endothelial cells throughout the brain and spinal cord after intraplantar injection of carrageenan [18]. Antiserum to interleukin-6 suppressed these responses [17]. However, little is known about which isoform of PLA_2_ is activated upstream of COX-2. Further studies are necessary to thoroughly determine which PLA_2_ isoforms are involved in prostaglandin synthesis in the central nervous system under inflammatory conditions.

## 5. Conclusion

The present study has indicated the importance of iPLA_2_ in prostaglandin synthesis at the inflammatory site and suggested that iPLA_2_ is a possible target for the treatment of inflammatory hyperalgesia.
